# Immunomodulatory biomaterials in HIV-1 infection prevention, control and treatment

**DOI:** 10.3389/fimmu.2025.1670423

**Published:** 2025-09-01

**Authors:** Jiaying Li, Huan Tang, Xiaoyi Zhou, Zijun Ni, Yuxin Liang, Xinyue Sun, Xun Zhuang

**Affiliations:** ^1^ School of Public Health, Nantong Third People’s Hospital, Affiliated Nantong Hospital 3 of Nantong University, Nantong University, Nantong, China; ^2^ Nantong Center for Disease Control and Prevention, Nantong, China

**Keywords:** immunomodulatory, biomaterials, HIV-1, T cell, macrophages

## Abstract

Acquired Immunodeficiency Syndrome (AIDS), caused by the Human Immunodeficiency Virus (HIV), is a global infectious disease that remains a significant global health challenge. Although antiretroviral therapy (ART) has significantly reduced HIV-1-related morbidity and mortality, it cannot eradicate viral reservoirs latent in host cells and long-term use of ART is also associated with issues such as drug toxicity, drug resistance, and poor patient compliance. Recent achievements in biomaterials have provided new ideas and tools for AIDS prevention, diagnosis, and treatment. Therefore, this review aims to summarize the latest research progress on biomaterials for immune cell functional regulation and immune activation strategies in HIV-1 prevention, control, and treatment. These approaches include enhancing the functions of CD8+ T cells and macrophages and synergizing with the targeted delivery and immunomodulatory capabilities of biomaterials to achieve viral clearance and immune reconstitution. Current challenges and the great potentials of biomaterials in drug delivery, vaccine development, and physical barriers for HIV-1 infection are discussed, along with future perspectives. By systematically reviewing relevant research findings, this review may provide theoretical basis and technical tools for promoting the clinical translation and application of biomaterials for HIV-1 infection.

## 1 Introduction

Acquired Immunodeficiency Syndrome (AIDS), caused by the Human Immunodeficiency Virus (HIV), is a global infectious disease that has profoundly impacted human health and social development since its first discovery in the 1980s (1, 2). Approximately 39 million people were living with HIV worldwide, including about 1.3 million new infections in 2022 (3). Although the widespread application of Antiretroviral Therapy (ART) has significantly reduced HIV-1-related morbidity and mortality, AIDS remains a major challenge in public health (4, 5). While ART effectively suppresses viral replication, it cannot eradicate viral reservoirs latent in host cells. Long-term use of ART is also associated with issues such as drug toxicity, drug resistance, and poor patient compliance (6–8). Additionally, high mutation rate of virus and immune evasion mechanisms limits the therapeutic efficiency of HIV-related vaccine or drugs (9–12).

Recent achievements in biomaterials have provided new ideas and tools for AIDS prevention, diagnosis, and treatment (13–15). Biomaterials are natural or synthetic materials with specific functions and biocompatibility, widely used in drug delivery, tissue engineering, immunomodulation, and other fields (16–19). In recent years, the application of biomaterials has made significant progress in HIV treatment. For example, materials such as nanoparticles, hydrogels, and microneedles have been used to develop targeted drug delivery systems, significantly improving the efficacy of anti-HIV drugs and patient compliance (20–22). Biomaterial-based vaccine delivery platforms have enhanced the immunogenicity of HIV antigens, offering new possibilities for vaccine development (10, 23, 24). Furthermore, the introduction of technologies such as biosensors and microfluidic chips has made rapid detection of HIV and viral load monitoring more convenient and precise (25–29).

This review aims to summarize the latest research progress on biomaterials for immune cell functional regulation and immune activation strategies in HIV-1 prevention, control, and treatment. These approaches include enhancing the functions of CD8+ T cells and macrophages and synergizing with the targeted delivery and immunomodulatory capabilities of biomaterials to achieve viral clearance and immune reconstitution. Current challenges and the great potentials of biomaterials in drug delivery, vaccine development, and physical barriers for HIV-1 infection are discussed, along with future perspectives. By systematically reviewing relevant research findings, this review may provide theoretical basis and technical tools for promoting the clinical translation and application of biomaterials for clearing HIV-1 infection.

## 2 Role of immune cells in HIV-1 treatment

The interaction between HIV-1 and the immune system ultimately leads to the loss of immune control against various pathogens (30). Immune cells play a crucial role in HIV-1 treatment. CD4+ T cells are the primary targets of HIV-1, and their depletion is a key factor in immune deficiency caused by HIV-1 infection (31, 32). In the early stage of HIV-1 infection, CD8+ T cells play an important role in controlling HIV-1 infection by recognizing and directly eliminating virus-infected cells. CD8+ T cells can kill infected cells by releasing granzymes and perforin, and inhibit viral replication by secreting cytokines such as interferon-γ (IFN-γ) (33, 34). Macrophages are one of the important target cells for HIV-1 infection. The virus can survive long-term in macrophages and continuously produce viral particles. However, activated macrophages can phagocytose and clear viral particles and infected cells, playing an important role in the early stage of HIV-1 infection. Additionally, as antigen-presenting cells, macrophages can present viral antigens to CD4+ T cells, activating immune responses (35). Follicular T helper (Tfh) cells are subset of CD4+ T cells in the B cell follicle of lymphoid tissues that facilitate B cells for affinity maturation, activation and germinal center (GC) formation, which has been regarded as one of the key replication sites of HIV. After HIV-1 infection, cell function of infected Tfh was dysregulated to cause B cell perturbations and led to insufficient neutralizing antibodies production (36). Tfr cells are another subset of CD4+T cells that expresses CXCR5 in the B cell follicle of lymphoid tissues, which inhibits Tfh functions and then regulates humoral immune response (37). Increased proportion of Tfr in the secondary lymphoid organs has been found in patients with chronic HIV infection, which further inhibits the maturation of antigen-specific responses with bNAb development (38). Dendritic cells (DCs) play a vital role in initiating adaptive immune responses by capturing and processing antigens from pathogens to activate CD8+ T cells, enhancing the immune system’s attack on HIV-1, however, it has been reported that cervical DCs can transport infectious HIV particles to the draining lymph nodes and spread HIV to susceptible T cells via cell-cell contact (39).

### 2.1 Role of T cells in HIV-1 treatment

T cells play an important role in HIV-1 infection. Regulatory T (Treg) cells function as inhibitors of effector T cell responses, helping to suppress autoimmune diseases and limit chronic inflammatory diseases (40). The proportion and functional changes of Treg cells are closely related to the progression rate of HIV-1 infection. Their excessive inhibitory effect may lead to the immune system’s inability to effectively control viral replication, accelerating disease progression (41). Treg cells suppress excessive immune responses by secreting anti-inflammatory cytokines, such as Interleukin-10 (IL-10) and transforming growth factor-β (TGF-β), to maintain immune homeostasis, but their immunosuppressive function also weakens antiviral immunity, promoting viral persistence and disease progression. During ART, Treg cells can reduce immune reconstitution inflammatory syndrome (IRIS), but their functional status also affects the efficacy of anti-HIV-1 strategies (42). For example, during the clearance of viral reservoirs, appropriate intervention in the regulatory role of Treg cells is needed to enhance the immune system’s ability to clear latent viruses (43). CD4+ T cells are the main target cells of HIV-1, and their massive depletion leads to immune deficiency (44, 45).

CD8+ T cells are considered the core effector cells of the anti-HIV-1 immune response (46). In the acute phase of HIV-1 infection, CD8+ T cells rapidly clear infected cells through cytotoxic mechanisms, reducing viral replication and spread (47). Based on cytotoxic mechanisms, CD8+ T cells secrete granzymes and perforin to penetrate the target cell membrane, form channels, allow granzymes to enter the target cell, and activate the intracellular apoptotic pathway, inducing programmed cell death of HIV-1-infected cells. In the chronic infection stage, CD8+ T cells may also inhibit HIV-1 replication through other non-cytotoxic mechanisms (48, 49). The antiviral factor secreted by CD8+ T cells is a diffusible lymphokine that can inhibit gene expression mediated by the HIV-1 long terminal repeat, thereby suppressing viral replication. CD8+ T cells can also secrete interferon-γ (IFN-γ), inducing cells to express MHC molecules, enhancing antigen presentation, activating macrophages, and inhibiting viral transcription and replication. Additionally, CD8+ T cells secrete β-chemokines, such as Macrophage Inflammatory Protein-1 alpha (MIP-1α), MIP-1β, and Regulated upon activation normal T cell expressed and secreted (RANTES), which can bind to the co-receptor C-C chemokine receptor type 5 (CCR5) required for HIV-1 entry into cells, preventing viral binding to receptors and inhibiting HIV-1 infection (50). Although CD8+ T cells play an important role in the anti-HIV-1 immune response, the high variability of the HIV-1 virus makes it difficult for CD8+ T cells to completely clear the virus (51). During chronic HIV-1 infection, continuous viral stimulation leads CD8+ T cells to enter a state of functional exhaustion, characterized by high expression of inhibitory receptors (such as programmed death 1 (PD-1), T cell immunoglobulin and mucin domain-containing protein 3 (Tim-3), etc.) on the cell surface (52). The high expression of these inhibitory receptors suppresses the activity of CD8+ T cells, reducing their killing ability and cytokine secretion, thereby weakening the immune system’s control over HIV-1. Therefore, how to sustainably and controllably activate CD8+ T cells is the key to treating HIV-1 infection through immune responses (53, 54).

### 2.2 Role of macrophages in HIV-1 treatment

Macrophages are another important target cell for HIV-1, playing a key role in all stages of HIV-1 infection. On the one hand, as Antigen-presenting cells, macrophages can present viral antigens to CD4+ T cells through the MHC-II pathway and activate CD8+ Cytotoxic T Lymphocytes (CTLs) through cross-presentation, thereby triggering immune responses in the early stage of HIV-1 infection. However, macrophages are also easily infected by HIV-1 due to their high expression of the CCR5 receptor and can survive long-term in tissues, such as microglia (a type of macrophage) in the brain, which can serve as long-term viral reservoirs, allowing the virus to persist in the body (55, 56).

Macrophages can be divided into M1 and M2 types based on their activation status. M1-type macrophages are activated in the early stage of HIV-1 infection, secreting proinflammatory cytokines (such as TNF-α, IL-1β, IL-6, etc.) and chemokines (such as CCL3, CCL4, CCL5), which help recruit other immune cells and enhance antiviral immune responses. In contrast, M2-type macrophages are more common in the chronic infection stage, mainly secreting anti-inflammatory cytokines (such as IL-10) to promote tissue repair, but this state may also facilitate viral latency and persistence. Therefore, how to controllably regulate macrophage polarization toward the M1 type to enhance antiviral immune responses is crucial (57).

## 3 Biomaterials for modulating immune cells in HIV-1 infection treatment

In recent years, treatment strategies for HIV-1 infection have continued to innovate, aiming to overcome the limitations of traditional ART and achieve more effective viral clearance and immune function recovery (58). Immune-activating biomaterials have been reported to promisingly regulate immune responses, through targeted delivery systems, sustained-release technologies, and immune activation functions, providing new strategies for conquering HIV-1 infection while delivering drugs (59). Sustained delivery and prolonged retention of antigens in lymph nodes can increase B cell and T helper follicular cell production followed by generating neutralizing antibodies to protect host cells from being infected by HIV (59). Boopathy et al. developed microneedles with silk fibroin, HIV ENV trimer and adjuvants to the sustained delivery of vaccine to effectively induce germinal center reactions to produce long-lasting B cells and plasma cells (60).

### 3.1 Targeted delivery systems

For HIV-1 infection treatment, while ART effectively suppresses viral replication, the short half-life of ART drugs requires patients to take medication frequently to maintain effective blood concentrations. This not only reduces medication compliance but also increases the complexity of treatment management. Meanwhile, ART drugs have significant systemic toxicity. For example, the non-nucleoside reverse transcriptase inhibitor Efavirenz often causes central nervous system adverse reactions and lipid metabolism disorders, severely affecting patients’ quality of life and long-term health (61). More importantly, existing therapies struggle to effectively target viral reservoirs—dormant viruses integrated into the host genome, which are prone to reactivation after treatment discontinuation, leading to disease recurrence (62).

Targeted delivery systems, leveraging the specific surface modification and molecular recognition capabilities of biomaterials, are constructed based on biomaterials such as nanoparticles, liposomes, and exosomes. By modifying specific ligands on their surfaces, such as CD4 antibodies, Arginine-Glycine-Aspartic Acid (RGD) and Ilexgenin A, these systems can achieve precise recognition and binding to virus-infected cells, enabling the accurate delivery of anti-HIV-1 drugs to infected cells. Preclinical studies have shown that integrase inhibitors using targeted delivery systems reduce viral load by 40% more than traditional formulations in animal models, with significantly decreased liver and kidney function damage, highlighting the great potential of biomaterials in precisely regulating drug distribution and release for HIV-1 treatment (63).

#### 3.1.1 Passive targeting systems

The passive targeting system is a delivery strategy that achieves specific enrichment based on the physical and chemical properties of drugs or carriers. Its core principle is to utilize inherent physiological and pathological characteristics in the body, such as differences in vascular permeability and tissue space size, to enable drugs to selectively accumulate in specific sites. Compared with active targeting, which relies on ligand-receptor recognition, passive targeting systems have become a highly promising drug delivery method in biomedicine due to their simple carrier design and low preparation cost (64).

Blood-Brain Barrier (BBB) penetration is a key application of passive targeting systems in central nervous system disease treatment. The BBB is composed of tightly connected brain microvascular endothelial cells, allowing only small molecules and lipophilic substances to diffuse passively, resulting in 98% of small-molecule drugs and almost all macromolecular drugs being unable to enter the brain (65). Polyethylene Glycol (PEG)-modified nanocarriers prolong blood circulation time by increasing steric hindrance and reducing macrophage phagocytosis, and achieve brain delivery through the transcytosis mechanism (such as transferrin receptor-mediated endocytosis) existing in the BBB (66). In HIV-1 treatment, this strategy can deliver protease inhibitors precisely to brain latent reservoirs. A study showed that PEG-modified PLGA nanoparticles increased the accumulation of the anti-HIV-1 drug Lopinavir in the mouse brain by 4.3 times, effectively inhibiting viral replication in the central nervous system (67).

Lipid Nanoparticles (LNPs) have become cutting-edge tools for passive targeting due to their unique physical and chemical properties. LNPs are typically composed of cationic lipids, helper lipids, cholesterol, and PEG-lipids, and can efficiently encapsulate mRNA or gRNA of the CRISPR-Cas9 system. Their particle size (approximately 60–100 nm) and surface charge characteristics enable them to be taken up by various cells through endocytosis. In HIV-1 treatment, LNPs encapsulating the CRISPR-Cas9 system achieved a 50% clearance rate of proviral DNA in mouse models. However, the passive targeting property of LNPs leads to non-specific distribution in organs such as the liver (68). Therefore, optimizing lipid composition and surface modification can further improve their targeting efficiency. Additionally, passive targeting systems can also utilize the high permeability of inflamed tissues to achieve drug enrichment. In chronic inflammatory sites caused by HIV-1 infection, the gaps between vascular endothelial cells increase, allowing nanocarriers to specifically accumulate. Studies have shown that PLGA-PEG nanoparticles of different sizes (131 nm, 312 nm, and 630 nm) were prepared by solvent dispersion, and their particle size distribution was optimized by differential centrifugation; larger nanoparticles (such as 596 nm) showed higher uptake efficiency in inflammatory cells. PEG-modified nanoparticles can interact with the adhesion coating in the inflamed colon, prolonging drug retention time and improving release efficiency, significantly enhancing drug concentration and therapeutic effect in inflamed sites (69). This property provides new ideas for treating HIV-1-related intestinal inflammation.

#### 3.1.2 Active targeting systems

The active targeting system is a novel technical system that endows drug delivery carriers such as nanoparticles with specific recognition and binding capabilities to target cells through surface functionalization modification. Compared with passive targeting systems that rely on physiological characteristics, it can deliver drugs to target sites more accurately and efficiently, greatly improving the therapeutic effect of anti-HIV-1 drugs (69, 70). The core principle of this system is to use the specific interaction between ligands and receptors, enabling drug-loaded nanoparticles to break through complex physiological barriers in the body and achieve precise targeting of HIV-1 infected cells, effectively reducing drug distribution in non-target tissues and lowering toxic side effects (66).

Targeting molecules such as CD4 antibodies can be modified on the surface of LNPs to precisely recognize and bind to HIV-1-infected CD4+ T cells. Modified LNPs can significantly enhance the enrichment efficiency of drugs in target cells, reduce effects on normal cells, enhance therapeutic effects, and lower systemic toxic side effects. Coupling specific antibodies against the HIV-1 envelope protein gp120 to the surface of liposomes can significantly enhance their targeting efficiency to CD4+ T lymphocytes (71). Additionally, exosomes, as natural nanoscale carriers, can be modified to load anti-HIV-1 drugs and specifically deliver them to infected cells due to their good biocompatibility and targeting properties, demonstrating excellent performance in inhibiting viral replication. Engineered exosomes with high expression of the scFv (a HIV-1-specific monoclonal antibody) on the surface and loaded with curcumin were developed to deliver anti-HIV agents for targeting and killing infected cells to suppress HIV-1 infection (72). Meanwhile, to overcome the side effects and development of drug-resistant HIV-1 by combination antiretroviral therapy, Shrivastava et al. developed an HIV-1 promoter-targeting Zinc Finger Protein to achieved effective repression on HIV-1 and further engineered exosomes loaded with RNAs encoding of this protein to suppress HIV-1 expression in mouse models for treating HIV-1 infection (73). Poly (lactic-co-glycolic acid) (PLGA), a synthetic polymer with good biocompatibility and degradability, is often used as a nanoparticle carrier. By adjusting the degradation rate of PLGA nanoparticles, long-acting sustained release of antiretroviral drugs can be achieved, effectively prolonging drug efficacy and reducing administration frequency. Chemically conjugating CD4 monoclonal antibodies to the surface of PLGA nanoparticles enables them to specifically recognize and bind to CD4 receptors on the surface of HIV-1 infected T cells. Such CD4 antibody-conjugated PLGA nanoparticles can increase drug concentration in HIV-1 infected cells by 3–5 times. The mechanism of action is that after the drug-loaded nanoparticles enter the bloodstream, the CD4 antibodies on their surface specifically bind to CD4 receptors on the surface of HIV-1 infected T cells, triggering endocytosis to promote nanoparticle entry into cells. Subsequently, the nanoparticles degrade in the cells, releasing drugs to exert antiviral activity (74).

Macrophages, as important viral reservoirs in the body, play a key role in viral persistent infection, spread, and immune escape (75). HIV-1-infected macrophages in the brain can cause neuroinflammation and neurodegenerative diseases, while lymph nodes are sites where HIV-1 is abundantly stored and replicated. Due to the high expression of mannose receptors on the surface of macrophages, mannose-modified liposomes can specifically recognize and bind to macrophages. Studies have shown that mannose ligands (such as Man-C4-Chol, Man3-DPPE) are conjugated to the surface of liposomes through chemical bonding (such as cholesterol or phosphatidylethanolamine groups) to achieve targeted delivery to macrophages. This enables drug delivery to tissues difficult to reach by ART, such as the brain and lymph nodes, providing a new effective approach to inhibit persistent HIV-1 infection (76).

In addition to antibody modification, peptide modification also shows great potential in targeting HIV-1-infected cells (77). Some peptides with specific amino acid sequences can efficiently bind to specific receptors on the surface of HIV-1-infected cells, guiding drug-loaded nanoparticles to target cells. For example, the Arginine-Glycine-Aspartic Acid (RGD) short peptide can specifically bind to integrin receptors overexpressed on the surface of HIV-1 infected cells, achieving efficient drug delivery through mediating endocytosis (78, 79).

As a natural product, Ilexgenin A has dual functional properties in targeting HIV-1 infected cells. It can not only regulate cell membrane fluidity and permeability, enhancing cellular drug uptake efficiency, but also specifically target Glucose Transporter 1 (Glut1), which is often highly expressed in HIV-1 infected cells (80). Further exploration of applying such natural products with targeting functions to anti-HIV-1 drug carrier design, combined with their unique biological activities, can develop more efficient and low-toxic anti-HIV-1 treatment strategies (81).

Additionally, aptamer-modified nanoparticles have gradually become an emerging tool in active targeting delivery. Aptamers are single-stranded nucleic acid molecules that can specifically bind to specific target molecules. By screening aptamers that specifically bind to surface markers of HIV-1 infected cells and modifying them on the surface of nanoparticles, precise targeting of HIV-1-infected cells can be achieved. Related studies have shown that an RNA aptamer targeting HIV-1 gp120 was modified with 2’-F and connected to an siRNA targeting the HIV-1 tat/rev region to form a complex (82). This complex can not only specifically bind to gp120 and enter cells expressing gp120 but also silence the corresponding tat/rev target gene, thereby potently and durably inhibiting HIV-1 virus replication in T cells (83).

#### 3.1.3 Stimuli-responsive systems

Stimuli-responsive systems are intelligent drug delivery systems that can sense changes in the body’s microenvironment (such as pH fluctuations, changes in specific enzyme concentrations, temperature differences, etc.) and trigger drug release accordingly. Compared with traditional drug delivery methods, this system can precisely release drugs at the lesion site, significantly improving the targeting and effectiveness of drug treatment, while reducing drug exposure in non-target tissues and minimizing toxic side effects (84). In the field of anti-HIV-1 treatment, stimuli-responsive systems show great application potential for the special microenvironment associated with HIV-1 infection.

pH-sensitive hydrogels, as a typical stimuli-responsive system, play an important role in preventing HIV-1 sexual transmission. The acidic environment (pH 3.8-4.5) of the female vagina provides a natural triggering condition for designing specific drug delivery carriers. pH-sensitive hydrogels are typically prepared from polymer materials containing acidic or basic functional groups. In neutral or alkaline environments, these functional groups are in a dissociated state, and the hydrogel remains swollen; when in the vaginal acidic environment, the functional groups are protonated, causing the hydrogel network structure to shrink, thereby promoting the release of encapsulated anti-HIV drugs (85). A pH-sensitive hydrogel based on acrylic acid and methacrylic acid have been developed. By loading the anti-HIV drug Tenofovir in such hydrogel system, more than 80% of the drug can be released within 24 hours in a simulated vaginal acidic environment, providing an effective tool for preventing HIV-1 sexual transmission (86).

Redox-responsive nanogels utilize differences in redox potential in different parts of the body to achieve precise drug release. Under normal physiological conditions, the glutathione (GSH) concentration outside cells is relatively low (about 2-20 μM), while in pathological microenvironments such as tumor tissues and inflammatory sites caused by HIV-1 infection, the GSH concentration can be as high as 10 mM, and the level of Reactive Oxygen Species (ROS) is significantly increased (87). Nanogels containing redox-sensitive chemical bonds such as disulfide bonds have been developed. In the normal physiological environment, the disulfide bonds remain stable, and the nanogel structure is intact; when entering the pathological microenvironment, the high concentration of GSH cleaves the disulfide bonds, causing the nanogel to degrade and release the encapsulated drugs (88). Although the direct application of redox-responsive nanogels in HIV-1 treatment is currently relatively limited, studies have delivered the cytokine IL-15Sa through such nanogels, successfully activating T cells in the tumor microenvironment and enhancing anti-tumor activity (89). This achievement may provide important inspiration for using redox-responsive nanogels to activate HIV-1-specific immune responses and clear HIV-1-infected cells in the future.

Additionally, enzyme-responsive delivery systems have gradually emerged in anti-HIV-1 drug delivery research. After HIV-1 infects host cells, it induces the production of some specific enzymes in the cells, such as HIV-1 protease (90). By introducing specific peptides (such as PQ) into hydrogels, these peptides can be recognized and cleaved by specific intracellular enzymes (such as Matrix Metalloproteinases MMP), thereby achieving precise drug release. This design enables nanocarriers to cleave under the action of specific intracellular enzymes after entering cells, triggering precise drug release and achieving efficient delivery of drugs into HIV-1-infected cells (91).

### 3.2 Long-acting sustained-release

Long-acting sustained-release based on biodegradable biomaterials can achieve precise control over the release rate and time of drug active ingredients (91). Compared with traditional administration methods, Long-acting sustained-release biomaterials have shown significant advantages in improving drug therapeutic efficacy and significantly improving patient medication compliance by prolonging the effective action of drugs in the body, reducing the administration frequency from multiple times a day to once every few months or even years, which reduces drastic fluctuations in drug concentration in the body and avoids toxic side effects caused by excessively high peak concentrations (92). After encapsulating antiretroviral drugs in PLGA microspheres, the long-acting drug release mechanism of subcutaneous implantation mainly relies on the biodegradable properties of PLGA. As a degradable polymer, PLGA gradually degrades through ester bond hydrolysis in the body, and drug release follows a biphasic kinetics: the initial rapid release (burst release) of surface drugs to quickly reach effective concentration, followed by continuous and slow diffusion release of drugs from the interior of microspheres as PLGA degrades from the surface to the interior, with the drug release time lasting for months. Additionally, the particle size, porosity, drug embedding method of microspheres, and local physiological environment (such as enzymes, pH) also affect the drug release rate, improving patient medication compliance by reducing administration frequency (93).

#### 3.2.1 Subcutaneous implantable biomaterials

Subcutaneous implantable biomaterials are an important application aspect of long-acting sustained-release materials. Silk fibroin (SF) is a natural polymer extracted from silk, consisting of a light chain (26 kDa) and a heavy chain (390 kDa), which are interconnected by disulfide bonds, which possesses excellent biocompatibility and biodegradation *in vivo* (13). As a natural polymer, chitosan hold good biocompatibility and bacteriostasis, which has been widely used in tissue engineering and drug sustained-release (24). Polycaprolactone (PCL), with its excellent biocompatibility, controllable degradation characteristics, and good mechanical strength, has become an ideal material for preparing subcutaneous implantable sustained-release devices (94, 95). After encapsulating Cabotegravir (CAB), an integrase strand transfer inhibitor, in a PCL sustained-release device and subcutaneously implanting it, it can achieve continuous and stable release of CAB for up to 6 months to effectively inhibit HIV-1 virus, which can maintain a stable blood drug concentration to avoid the problems of large blood drug concentration fluctuations and frequent administration for improving patient medication compliance (96). Subcutaneous implantable biomaterials not only greatly reduce the medication burden on patients but also effectively reduce the risk of viral drug resistance by maintaining stable blood drug concentrations.

#### 3.2.2 Microneedle patch

Microneedle patches, prepared by mixing drugs with biodegradable materials, has opened up a new path for the long-acting prevention and treatment of HIV-1, which achieves transdermal drug delivery through dissolvable microneedle arrays, which gradually dissolve after penetrating the epidermis, releasing drugs into the body. Studies have shown that a dissolvable microneedle array patch (MAPs) loaded with Elvitegravir for HIV-1 prevention and treatment was developed using a double casting method (97). *In vitro* and animal experiments showed that the microneedle patch has high structural strength, can penetrate the skin by 580 μm and dissolve within 2 hours and delivery efficiency of Elvitegravir is about 40%, and it can continuously release drugs for 3 months in rodents, providing a minimally invasive and long-acting new solution for HIV-1 treatment (98). Microneedle patches penetrate the stratum corneum of the skin through microneedles, delivering drugs directly into the epidermis or upper dermis, thereby bypassing the skin barrier and enabling drugs to quickly reach the site of action and produce therapeutic effects. This delivery method avoids the first-pass effect of the liver and gastrointestinal degradation, improving drug bioavailability (99). Compared with traditional injection administration, microneedle patches have significant advantages of being painless, easy to operate, and highly acceptable to patients, which can also avoid the risk of needle stick infection, showing great application potential in the field of HIV-1 Pre-Exposure Prophylaxis (PrEP) (100).

## 4 Future perspectives

After HIV-1 infection, the virus can distribute in various tissues and organs. Due to the different extracellular matrix structures and cell types in each tissue, how to effectively and precisely deliver drugs to target cells remains a challenge in HIV-1 infection treatment. The development of nanomaterial-integrated drug delivery systems, gene editing, and immune-activating functional biomaterials, as well as photothermal/magnetic-controlled nanocarriers for spatiotemporally controlled release, will significantly improve the treatment effect of HIV-1 infection. Biomaterials based on nanotechnology have been reported to boost CD8+ T cells as HIV vaccines, such as enhancing the cross-presentation of delivered viral antigen and co-delivery of the antigen and adjuvant composition. Xu et al. developed a virus-like fullerenol NPs as a HIV-1DNA vaccine, which possessed dual functions as plasmid DNA carrier and activator of host immunity to induce robust CD8+ T cells-mediated cellular immunity (101). Aline et al. investigated a PLA nanocarriers-based HIV vaccine containing HIV p24 protein, and used this nanovaccine to stimulate DCs and then adoptively injected the pulsed DCs into human. They found that the nanovaccine significantly increased the costimulatory molecules expression and elicited strong Th1/Th2-type cytokines secretion of DCs, ultimately activating the systemic CTLs against HIV (102). However, the toxicological risks of biomaterials or nanocarriers cannot be ignored. For example, carbon nanotubes may trigger inflammation. It is necessary to develop degradable materials (such as polyesters), natural carriers (such as extracellular vesicles), or natural polymer materials to reduce immune rejection and biological toxicity. Meanwhile, the complex preparation processes and high production costs of multifunctional biomaterials are also bottlenecks to break through for industrialization and large-scale clinical translation. Additionally, due to the numerous subtypes of the HIV-1 virus, how to target HIV-1 viral molecules is also a challenge. Bacterial and fungal infections are common complications of HIV-1 infection. Meanwhile, How to achieve collaborative prevention and control of multiple pathogens remains a challenge. Incorporating antibacterial and antifungal components into biomaterials can prevent the co-transmission of HIV-1 and other sexually transmitted infections.
